# Advances in Electrospun Hybrid Nanofibers for Biomedical Applications

**DOI:** 10.3390/nano12111829

**Published:** 2022-05-27

**Authors:** Viraj P. Nirwan, Tomasz Kowalczyk, Julia Bar, Matej Buzgo, Eva Filová, Amir Fahmi

**Affiliations:** 1 Faculty of Technology and Bionics, Rhine-Waal University of Applied Science, Marie-Curie-Straβe 1, 47533 Kleve, Germany; virajpratap.nirwan@hochschule-rhein-waal.de; 2 Institute of Fundamental Technological Research, Polish Academy of Sciences (IPPT PAN), Pawinskiego 5B, 02-106 Warsaw, Poland; tkowalcz@ippt.gov.pl; 3 Department of Immunopathology and Molecular Biology, Medical University, Bujwida 44, 50-345 Wroclaw, Poland; julia.bar@umw.edu.pl; 4 BIOFABICS, Rua Alfredo Allen 455, 4200-135 Porto, Portugal; matej.buzgo@biofabics.com; 5 Department of Tissue Engineering, Institute of Experimental Medicine of the Czceh Academy of Sciences, Vídeňská 1083, 14220 Prague, Czech Republic; eva.filova@iem.cas.cz

**Keywords:** hybrid nanofibers, electrospinning, nanoparticles, functional agents, tissue engineering, nanomedicine, drug delivery, bone regeneration

## Abstract

Electrospun hybrid nanofibers, based on functional agents immobilized in polymeric matrix, possess a unique combination of collective properties. These are beneficial for a wide range of applications, which include theranostics, filtration, catalysis, and tissue engineering, among others. The combination of functional agents in a nanofiber matrix offer accessibility to multifunctional nanocompartments with significantly improved mechanical, electrical, and chemical properties, along with better biocompatibility and biodegradability. This review summarizes recent work performed for the fabrication, characterization, and optimization of different hybrid nanofibers containing varieties of functional agents, such as laser ablated inorganic nanoparticles (NPs), which include, for instance, gold nanoparticles (Au NPs) and titanium nitride nanoparticles (TiNPs), perovskites, drugs, growth factors, and smart, inorganic polymers. Biocompatible and biodegradable polymers such as chitosan, cellulose, and polycaprolactone are very promising macromolecules as a nanofiber matrix for immobilizing such functional agents. The assimilation of such polymeric matrices with functional agents that possess wide varieties of characteristics require a modified approach towards electrospinning techniques such as coelectrospinning and template spinning. Additional focus within this review is devoted to the state of the art for the implementations of these approaches as viable options for the achievement of multifunctional hybrid nanofibers. Finally, recent advances and challenges, in particular, mass fabrication and prospects of hybrid nanofibers for tissue engineering and biomedical applications have been summarized.

## 1. Introduction

**Figure 1:**
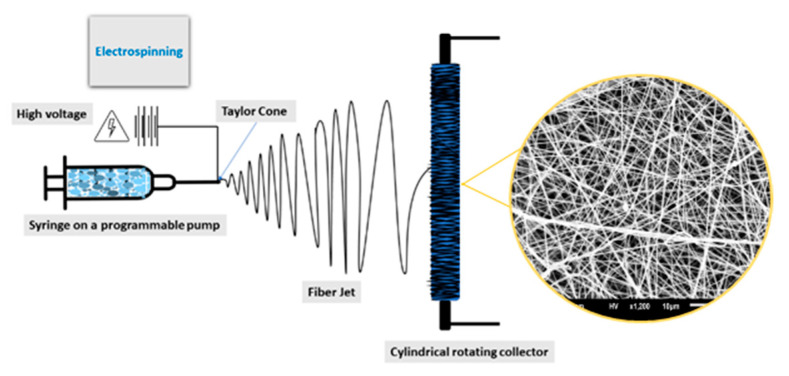
Schematic representation of simple electrospinning set up with rotating cylinder collector.

Electrospinning has emerged as a principal technique for the fabrication of 3D nanofibers. This technique has seen enormous growth in the last decade, leading to a generation of comprehensive research, demonstrating effective implementation of electrospinning (Figure 1) to fabricate nanofibers [1,2,3,4,5]. Recently, this technique has garnered attention for the fabrication of nanocompartments from a wide variety of materials, with a focus on the development of substitute materials capable of supporting tissue regeneration, wound dressing, support bone regeneration, and filtration, among others [6,7,8,9,10,11]. While the possibility of supporting an extensive range of applications presents electrospinning as an attractive tool, increasingly restrictive physiological and structural requirements for biological applications has led to the development of constraints. For instance, electrospun nanofibers must possess characteristics such as porosity, conformity, and interconnected architecture, and mechanical, thermal, and electrical properties [5,12]. Additionally, biocompatibility and biodegradability are other important characteristics, which play significant roles in biomedical applications. Here, these properties can influence cell proliferation, adhesion, toxicity, and growth behavior while minimizing a negative effect on cell growth [13,14,15,16,17,18,19]. Therefore, effectively capturing those physiological and structural requirements requires optimization of a range of complex electrospinning parameters and demand the use of materials with tailored properties [20,21,22]. 

### 1.1. Coelectrospinning to Improve Electrospinnability

The fabrication of nanofibers using electrospinning requires the fulfilling of certain conditions. Significantly, it requires the processability of the polymers, including their stability in commonly used solvents, molecular weight etc. [23,24]. These properties are relatively easy to compensate in traditional synthetic polymers, which possess very long polymer chains and the ability to form relatively stable solutions in a wide range of solvents, making them ideal for electrospinning. Applications such as sensors, stimuli response, air purification, water filtration, catalysis, energy harvesting, and electronics have benefitted substantially from nanofibers fabricated using those polymers [24,25]. However, polymers that are ideal for overcoming restrictive requirements of biomedical applications generally have poor electrospinnability [26]. For instance, naturally occurring polymers such as collagen, gelatin, chitosan, fibrinogen, and silk fibroins exhibit a weak electrospinning ability. They have poor mechanical stability, high degradability, low solubility, and tend to form gel at high concentrations [26,27,28,29]. Nonetheless, by blending these polymers with synthetic or other natural biopolymers, it is possible to overcome their electrospinning drawbacks. This process of blending two polymers has often been referred to as coelectrospinning. It allows for the improvement of the electrospinnability of one polymer by the introduction of assisting polymers, generally with very high molecular weights, better mechanical properties, solubility, and viscosity [30,31]. Here, coelectrospinning provides the possibility of incorporating dissimilar materials and the fine-tuning of unique characteristics in fabricated nanofibers, while increasing the processability of a wide range of biocompatible polymers. It results in the fabrication of nanofibers as nanostructured scaffolds mimicking microporosity, intricate morphology of extracellular matrix (ECM), and the possession of beneficial characteristics for tissue healing, tissue growth, disease theranostics, etc. [32,33,34,35,36,37]. Numerous scaffolds made of synthetic/natural polymers and their hybrids have been developed for these applications. For application in biological systems, these scaffolds provide structural and chemical moieties mitigating many issues encountered while using traditional materials. Further, blending and coelectrospinning approaches allow fine-tuning of structural, mechanical, and biological properties of materials. It provides the possibility of obtaining nanofibers from naturally occurring biodegradable polymers such as chitosan, lignin, and silk fibroin which can support cell growth and adhesion and minimize biocompatibility issues [10,38,39]. However, the fabrication of nanofibers from naturally occurring polymers remains a huge challenge, and the blending of these naturally occurring polymers with synthetic polymers often tends to dilute the advantages [40,41,42].

### 1.2. Multifunctional Coelectrospun Nanofibers via Immobilization of Functional Agents

The characteristics of nanocompartments obtained simply from polymer blends are still restrictive, and there is a lot to be desired when it comes to their mechanical, electrical, or optical properties. The improvement of these properties through the immobilization of various functional agents could provide a multifunctional characteristic to nanofibers. These functional agents come in many forms and include inorganic/organic nanoparticles (NPs), drug molecules, perovskites, biomolecules such as proteins, dendrimers, carbohydrates, lipids, growth factors, and hormones, among others [24,43,44,45]. For instance, coelectrospinning allows for the inclusion of naturally occurring minerals, such as hydroxyapatite, playing an important role in the development of scaffolds for bone regeneration. The possibility of including functional agents in nanofiber matrices offers an improvement in both structural and biological capabilities, which is vital for tissue-engineering applications [46,47,48]. Undoubtedly, the immobilization of diverse functional moieties in coelectrospun nanofiber matrices provides huge improvements in both mechanical and physiochemical properties. 

### 1.3. Functionalization Using NPs

**Figure 2:**
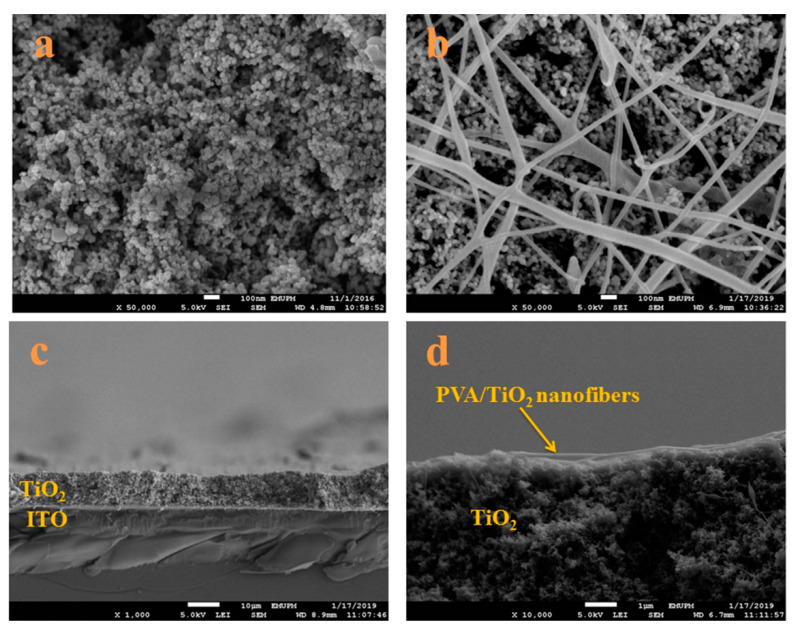
FESEM image of (**a**) TiO_2_ nanoparticles, (**b**) TiO_2_ nanoparticles with PVA/TiO_2_ nanofibers, (**c**) cross-sectional view of TiO_2_ nanoparticle, and (**d**) a cross-section of TiO_2_ nanoparticles with PVA/TiO_2_ nanofibers. Reproduced from Ref. [64].

The immobilization of nanoparticles is one of the most promising methods to provide nanofibers additional functionality and improved mechanical and physicochemical properties [49,50,51,52,53,54,55]. A remarkable amount of research has already established the advantages of nanoparticles as advanced materials with unique characteristics such as a plasmonic effect, superparamagnetic effect, and photochromatic effect, among others [56,57,58,59,60]. NPs have been used to enhance efficiency and specificity in drug delivery, imaging, labelling, and sensing applications. In addition to improving physicochemical properties of nanofibers, NPs also play a significant role in cell adhesion, proliferation, and growth behavior [61,62,63]. For instance, titanium dioxide NPs have been used to decorate organic nanofibers to achieve a unique, hybrid nanofiber [64,65]. This unique combination provided an organic part acting as an electron-rich domain as a donor, whereas inorganic TiO_2_ accepts electrons and facilitates their transfer across the membrane (Figure 2). 

Additionally, nanofibers with various functional agents have the advantage of possessing selectivity, which is useful for identifying single or various filtrates from a source of pollutants. Therefore, it could be highly effective for sensing and filtration. Especially in case of filtration, nanofiber filters are a huge step forward from traditional filters, owing to the presence of a large number of nanosized pores and their capacity to filter out even microbes with a high efficiency. These nanofiber filters have been shown to function optimally while mitigating issues such as high pressure drops, large pore sizes, and charging effects. Moreover, nanofiber filters have been used to replace reverse osmosis membranes, thereby lowering the amount of energy required during the filtration process [66,67,68,69]. The immobilization of functional agents, such as AuNPs, for optical sensing applications can provide a quick and effective method of portable and efficient sensors. Furthermore, it is possible to include both a sensing and filtering capability by inducing multiple functional agents [70]. Co-electrospinning also offers the possibility of supplementing nanofibers made from synthetic polymers such as nylon and polyacrylonitrile (PAN) with antimicrobial agents, reducing the accumulation of harmful microbes and providing additional protection [71,72]. NPs such as Ag and Cu have shown promising results as antimicrobial agents for applications such as wound dressing or face masks [73,74,75,76,77]. Superparamagnetic iron oxide (SPIONs) NPs have been immobilized in nanofibers to develop a platform for cancer-cell hyperthermia, bioimaging markers, and drug carriers [78,79,80,81]. AuNPs, owing to their plasmonic, antimicrobial effect and low bio-toxicity, have assumed prominent roles in biomedical applications such as biosensors and drug-delivery agents, as well as in photothermal therapy and imaging [40,82,83]. As mentioned earlier, hydroxyapatite is another example of functional NPs which has been shown to induce osteoblastic differentiation in cells and improve structural and chemical properties of nanofibers [48,84]. Si nanoparticles are significant bioactive functional agents immobilized on nanofibers. Si nanoparticles have been shown to be biocompatible and biodegradable with a potential to be used in a theranostics modality [40,85,86]. 

#### Pulsed Laser Ablation—Clean Alternative for Synthesis of NPs as Functional Agents

The functionalization of nanofibers, especially using chemically synthesized NPs, presents a huge challenge in terms of biotoxicity due to the presence of impurities or unreacted chemicals, ligands, or stabilizing agents. These impurities can interfere with characteristics of functional agents and are toxic for biomedical applications [87,88,89]. Traditional synthesis routes require surface modifiers (ligands) to control the size, morphology, and stability of NPs. These ligands can interfere with hormonal and signal mechanisms with adverse effects. Additionally, processing steps are always required for purification and for organic solvents which are cytotoxic, restricting the transition to NPs despite their novel properties [87]. Laser ablation has emerged as an alternative method to overcome these issues [90,91,92]. Pulsed laser ablation in liquids (PLAL) utilizes pulsed laser radiation to ablate a solid target in liquid. Briefly, a highly energized, pulsed femtosecond (fs) laser is directed toward a target which is either the bulk material or in powdered form. The laser is focused via a lens on the top of the target, generating an ionized species inside a liquid medium, eventually forming nanoclusters and leading to the formation of a colloidal nanoparticle solution [90,93,94,95]. NPs formed by this method have a unique chemistry, resulting from the bare surface of those particles, and it is easier to isolate them. Here, nanoparticles remain stable in the absence of ligands or general stabilizing (CTAB) agents, which common in chemical synthesis routes. PLAL utilizes a medium such as deionized water, excluding chances of contamination, and leads to the formation of highly stable, low size-dispersed NPs. Therefore, PLAL has emerged as an effective alternative for the fabrication of NPs used as functional agents in nanofibers [82,96,97,98,99,100,101,102]. 

### 1.4. Biofunctionalization of Nanofibers

Other than NPs, functional agents such as inorganic polymers and biomolecules have been used to improve characteristics of nanofibers. Biofunctionalization refers to the immobilization of such biomolecules or biomass in nanofibers’ matrix. Nanofibers have been biofunctionalized to include agents which are antioxidants, anti-inflammatory, antibacterial, antifungal, vitamins, etc. [103,104,105]. Moreover, biofunctionalization can also improve biocompatibility and bioactivity of nanofibers. Nanofiber scaffolds used for tissue regeneration can be modified to provide the capability of offering nutrition, antimicrobial activity, and structural support [106,107,108,109,110]. Drug molecules are another class of functional agents, which have been added to nanofibers through co-electrospinning. These nanofibers are vital in the development of efficient drug-delivery systems (DDS). Such systems have the benefit of modulating the release of the drugs, and possess selectivity by targeting a desired area with efficiency. DDS promises to harmonise the drug-delivery mechanism while reducing potential side effects [111,112]. The separation of biomolecules can be performed efficiently by tapping on the surface properties of nanofibers and by immobilizing the molecules, which selectively bind to ligands. Through selective chemical interaction with target molecules, these co-electrospun hybrid nanofibers can isolate them effectively [113,114]. 

### 1.5. Perovskites as Functional Agents

Perovskites have been attracting a lot of interest as another vital mineral functional agent in nanofibers. Primarily, this mineral is in demand for solar cell technology due its electrical, magnetic, and tunable luminescent properties [115,116,117,118]. Recently, they have been included as functional agents in nanofibers for antibacterial and biomedical applications. Gora et al. has shown a successful modification of perovskites with Ag to obtain NPs, which they have immobilized in nanofibers. The resulting nanofibers possessed effective antimicrobial properties and improvements in diameter and tensile strength [119]. Nanofibers that have been fabricated with modified or unmodified perovskites immobilized in their matrix provide a new class of luminescent nanomaterials. Their promising applications can be found through the modulation of emissive properties, stability against UV radiation, use as a stretchable/bendable optically active material, and biosensing applications, among others [120]. 

**Figure 3:**
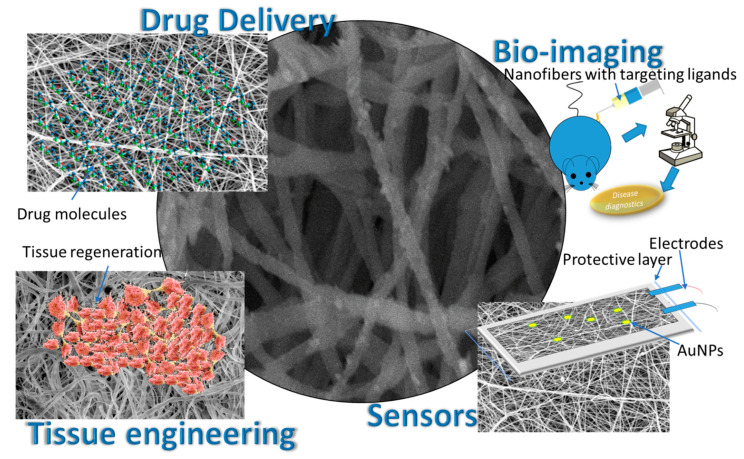
Common biomedical applications of functionalized nanofibers.

Here, some of the latest achievements based on remarkable improvements and new developments in electrospinning are presented from recent publications (Figure 3). Additionally, we have provided an overview of their prospective applications in the field of tissue engineering and drug delivery and biosensing with some observations on prospects. 

## 2. Electrospinning of Polymers with Limited Spinnability by Templating and Functionalization

One of the advantages of electrospinning is that it allows a wide of range of polymers to be fabricated into nanofibers. It is possible to obtain, from the literature, a comprehensive list of synthetic and natural polymers which have been electrospun for a variety of applications [121]. Generally, synthetic polymers have good electrospinnability, specifically due to their higher molecular weights and dissolution in a wide range of solvents. Naturally occurring polymers used as green alternatives for electrospinning bring excellent biocompatibility and degradability, but present many challenges during electrospinning. Chitosan is one of such biopolymers, a biodegradable polymer derived from shells of arthropods, such as crustaceans and insects. Until recently, it was often a by-product with low economic value. However, there has been a rise in the development of the product, especially in biomedicine where chitosan or its derivatives have been used [122,123]. Chitosan has many attractive properties such as biocompatibility, dissolution in water, and antimicrobial and antifungal properties which support its use in the development of new biomaterials [124,125,126]. However, difficult processability, lower molecular weight, and a tendency to form gel at higher concentrations make it unsuitable for electrospinning [127]. Chitosan dissolubility could be improved in dilute organic acid (formic acid/acetic acid) solutions, either in aqueous or organic solvents, including ethanol, methanol, and acetone. Even then, it has the tendency to form a 3D-intranetwork due to strong hydrogen bonds, and possesses poor stretchability under an electric field [128]. Therefore, it is often co-blended with another polymer to improve electrospinnability [40]. The resulting co-blended nanofibers display an improvement in physicochemical, mechanical, and biological properties. Adeli et al. has demonstrated that blending chitosan with polyvinyl alcohol (PVA) and starch improved its electrospinnability. Here, PVA acted as a co-spinning agent while the presence of starch improved the water absorption capacity of nanofibers. The stability of nanofibers in an aqueous solution was further improved by cross-linking using glutaraldehyde. Nanofiber mats obtained through this process were effective against both gram-negative and gram-positive bacteria. Moreover, it provided a porous surface for wound breathing and captured exuded material [129]. In another study, Yang et al. used similar a principle to obtain nanofibers from chitosan. The group utilized polyethylene oxide (PEO), which is a biodegradable and biocompatible polymer, easily dissolved in polar and nonpolar solvents alike [130]. An equivalent weight ratio of polymers was dissolved in dilute acetic acid solution and electrospun. Uniform, bead-free nanofibers were obtained as a result, which were then processed and grated with poly(glycidyl methacrylate) (PGMA) and polyethyleneimine (PEI) to improve the metal-ion-adsorption capabilities of nanofibers and stability in an aqueous medium. The selective adsorption of nanofibers in Cr, Cu, and Co ion mixtures showed that the presence of protonated amine groups on the surface of nanofibers were effective in removing negatively charged species of metal ions from the solution. Further, the effectiveness of absorption increased in slightly acidic environments. Such an approach could provide a platform for environmentally friendly and effective methods for the filtration of heavy metal ions at water treatment facilities. However, the optimization of nanofiber yields and homogeneity in nanofiber composition require further research.

Based on a similar strategy, chitosan-blended nanofibers were fabricated using PEO with a molecular weight of 300 kDa as a co-spinning agent. PEO is an excellent candidate for electrospinning due to its high molecular weight, ability to solubilize in a wide range of solvents, biodegradability, and good biocompatibility [74,131]. Moreover, bare laser ablated gold nanoparticles (AuNPs) were used as functionalization agents which offered a unique, ligand-free, and uncontaminated surface. These AuNPs also offer the absence of interference of stabilizing ligands, possessing excellent surface plasmon, photothermal, and antimicrobial properties, and can be used for applications such as photothermal therapy, bioimaging, and biosensors. Bare laser ablated AuNPs were obtained by focusing a femtosecond laser (Yb:KGW laser, Amplitude Systems, 1025 nm, 480 fs, 1 kHz) on a solid target in deionized water (as shown in ref. [85,132]). 

**Figure 4:**
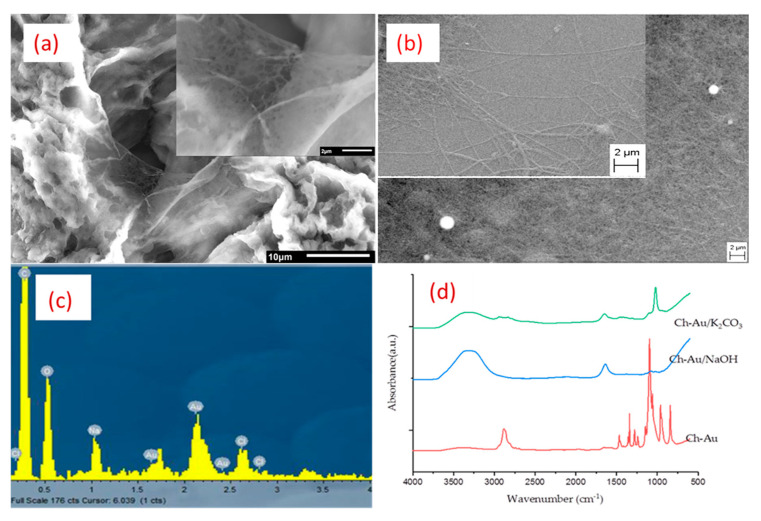
SEM micrograph of AuNPs-functionalized Chitosan (PEO) nanofibers neutralized: (**a**) with 1M K_2_CO_3_ in 70% ethanol; (**b**) with 5M NaOH in methanol; (**c**) corresponding EDX spectroscopy graph showing presence of AuNPs after neutralizing with NaOH method; (**d**) FTIR spectra of nanofibers functionalized with AuNPs before neutralization (Ch-Au) and after neutralization with K_2_CO_3_ (Ch-Au/K_2_CO_3_) or NaOH (Ch-Au/NaOH). Adapted from Ref. [132].

Further, neutralization strategies to improve the stability of nanofibers in an aqueous medium were compared (for details, see ref. [132]). Subsequently, pristine nanofibers were obtained with uniform morphology and absence of beads, possessing an average diameter of 189 nm ± 100 nm. Functionalized nanofibers showed a cylindrical morphology, without the presence of beads, and their average diameter was 189 nm ± 86 nm. Thermal analysis showed that AuNPs in nanofibers appear to act as heating spots, which is favorable for photothermic applications. Finally, the improvement of the stability of nanofibers in aqueous solutions was performed by dipping them in 1M K_2_CO_3_ in 70% ethanol and 5M NaOH in 70% methanol. Neutralization with 5M NaOH was effective in stabilizing the nanofibers while preserving their micro- and nanostructure. Whereas neutralization with 1M K_2_CO_3_ gave stable scaffolds, their nanostructure could not be preserved. Further, EDX analysis of fibers after NaOH treatment confirmed the presence of AuNPs. FTIR analysis also showed no observable characteristic peaks of PEO in treated nanofibers, while characteristic peaks of chitosan were still observable. This method proved to be effective in obtaining stable chitosan nanofibers (Figure 4). However, their effect on the biological system remains to be seen. Further, the ratio of chitosan should be increased to augment its effect as a scaffold in a tissue-engineering platform. 

Felipe et al. had also electrospun chitosan using PEO as co-spinning agent. The nanofibers were functionalized with carboxymethyl-hexanoyl chitosan/dodecyl sulphate nanoparticles loaded with pyrazoline H_3_TMO_4_ for skin cancer treatment [133]. Polymers were dissolved in an aqueous solution, limiting the toxicity effect, which might have occurred due to the use of organic solvents. The group successfully obtained nanofibers with a narrow diameter dispersion and a homogeneous distribution of functional agents. Moreover, an in vitro study by the group showed that the encapsulation of the drug in nanofibers promoted their slow and sustained release, with cytotoxic behavior towards mouse melanoma cell lines. Here, the group not only successfully electrospun chitosan but also demonstrated the effectivity of electrospun nanofibers as drug-delivery models for localized cancer treatment. 

Similarly, inorganic polymers, which are an interesting class of materials containing highly electronegative domains such iron, phosphorous, and boron in their backbone, can be spun into nanofibers by co-electrospinning [134]. The presence of these domains provides inorganic polymers with unique properties such as conductivity, the ability to organize in a systematic manner for use in optoelectronic devices, high thermal stability, and good solubility [135,136,137]. The manifestation of these polymers as nanofibers would amplify their characteristics and provide a large surface area for their activation and activity. Therefore, poly(ferrocenylmethylphosphinoborane) Fe A and poly(ferrocenylphosphinoborane) Fe B, which possess these domains in their backbone which is valence–isoelectronic with a C-C backbone found in general organic polymers, were chosen to be subjected to coelectrospinning into nanofibers [138]. Possessing such attractive properties could open a wide avenue of applications for these polymers, including catalysis as charge transferring modalities and thermally stable materials in the form of hybrid nanofibers. However, the low molecular weight of these polymers hinders their electrospinnability. PEO and polystyrene (PS) were excellent polymers with a high molecular weight and electrospinnability in compatible solvents and were ideal to be used as co-spinning agents. 

Using the co-spinning method, long, cylindrical, homogeneous nanofibers without beads were fabricated from blends on smart, inorganic polymers Fe A, Fe B, and PEO/PS. Nanofibers exhibited an average diameter ranging from 623 nm to 478 nm for Fe A/PS and Fe B/PS blends, respectively. The average diameter of nanofibers, prepared from the solution blended with PEO, had an average diameter of 525 nm and 491 nm for Fe A/PEO and Fe B/PEO blends, respectively [139]. Thermal analysis using TGA showed that pristine inorganic polymers had remarkable thermal stability, especially for nanofibers containing Fe A/PS and Fe B/PS blends. 

These studies demonstrate the effectivity of co-electrospinning approach to obtain nanofibers from highly desirable polymers such as chitosan, which inherently have low electrospinnability. Using the co-electrospinning approach has resulted in the fabrication of environmentally sustainable nanofibers with good biocompatibility for applications such as wound dressing materials with hydrophilicity, water retention, and antimicrobial capabilities. Additionally, it has established nanofibers as a platform for modulated drug-delivery systems, localized cancer treatment, photothermal therapy, catalysis, and sensors, among others. 

## 3. Fabrication of Inorganic NPs Functionalized Nanofibers

Nanofibers functionalized with inorganic nanoparticles provide a combination of unique properties with a very large surface area to volume ratio. The immobilization of nanoparticles on nanofibers can be achieved by either solubilizing the nanoparticles directly in an electrospinning solution [140,141], reducing the precursor for nanoparticles in situ, growing them on nanofibers [142,143], or by electrospraying/spin coating nanoparticles on fibers’ surfaces [144,145]. 

While designing biomaterials for biomedical applications, there are many structural, physical, chemical, and biological challenges to be overcome. The engineering and growth of tissues require a complex environment difficult to emulate [146,147,148]. Hybrid nanofibers encapsulating nanoparticles is one way of improving the properties of scaffolds. By solubilizing the nanoparticles directly in an electrospinning solution approach, Leones et al. fabricated nanofibers using polycaprolactone (PCL) as a matrix immobilized with various inorganic/organic nanoparticles. They managed to obtain stabilized electrospinning containing Ag, SiO_2_, cellulose nanocrystals, and hydroxyapatite at 1% (*w*/*v*) with respect to the polymer [149]. The resultant nanofibers showed properties vastly improved compared to bulk materials. In the mechanical analysis, the flexibility of materials increased with an increasing diameter and by the addition of nanoparticles. In another study, Yang et al. had obtained AgNPs and ciprofloxacin immobilized janus nanofibers with two matrices, poly(vinyl pyridine) and ethyl cellulose, for wound dressing application. With the objective of combining the hybrid antibacterial effect of AgNPs and the drug ciprofloxacin in the same material, the group had modified their approach by solubilizing the nanoparticles in their respective matrices. Using the acentric type of spinneret, hybrid janus nanofibers were electrospun. This type of set-up provided a dual mechanism to the nanofibers where the drug was effective for quick action with 90% of the drug being released in 30 min, while the presence of AgNPs provided a sustained antimicrobial effect [150]. This approach offers a solution for applications where a hybrid two-step drug release is required. Similarly, the development of separators, especially for lithium ions batteries, have benefitted from the use of nanofibers containing inorganic functionalizing agents. While the polymer matrix could help isolate electrolytes and prevent electrical short-circuits, the inorganic part could sustain the charge transfer in addition to providing thermal/mechanical stability. Additionally, the presence of inorganic nanoparticles can improve electrochemical stability [151]. Jaritphun et al. had recently demonstrated the benefits of using such structures as separators. They had achieved this by the fabrication of hybrid nanofibers with inorganic nanoparticles sandwiched between two layers. Here, immobilization of nanoparticles on the surface of a nanofiber was performed by electrospraying [152]. Additionally, hybrid nanofibers contained a layer of SiO_2_ and Al_2_O_3_. These hybrid nanofibers had higher thermal stability and demonstrated improved cycling performance. Moreover, the presence of inorganics enhanced the mechanical properties of fibers as a scaffold and their wettability, leading to efficient performance. 

**Figure 5:**
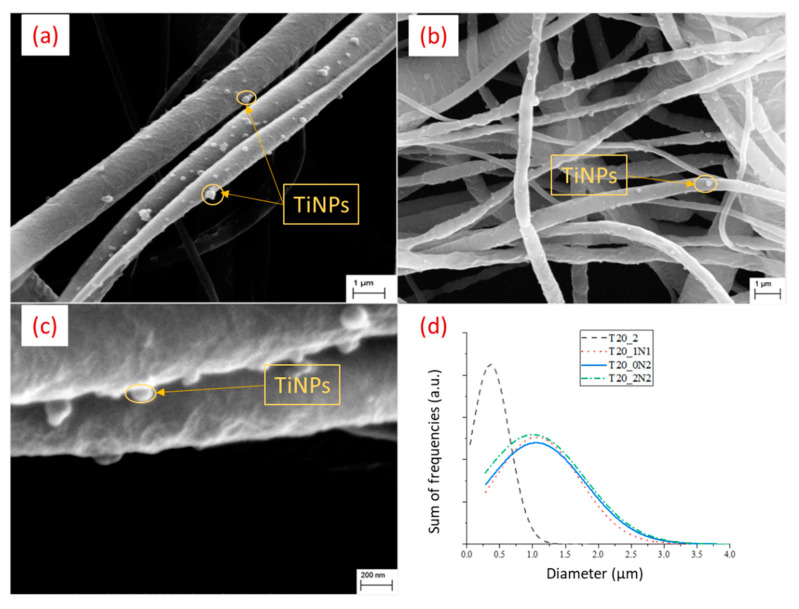
Ligand-free TiN NPs-functionalized PCL (20% *w*/*v*) nanofibers with various concentrations of TiN NPs in electrospinning solutions: (**a**) 1 mL (0.15 mg L^−1^), T20_1N1; (**b**) 2 mL (0.15 mg L^−1^), T20_0N2; (**c**) 2 mL (0.45 mg L^−1^), T20_0N6; (**d**) statistical analysis of nanofibers’ diameter measured using ImageJ. Adapted from Ref. [157].

Recently, an effective immobilization of inorganic (titanium nitride) nanoparticles in a PCL matrix was demonstrated (Figure 5). Here, laser ablated ligand-free titanium nitride nanoparticles (TiN NPs) were used as functional agents. The NPs have emerged as an excellent modality for applications such as photohyperthermia, as their absorption spectra lie in the range of 640–720 nm (biological transparent window). Still, thanks to the laser ablation methods, their unique surface chemistry promises an unhindered photothermal effect [99,153]. To mitigate biocompatibility and biodegradability issues, PCL was a natural choice of polymer for the fabrications of scaffolds supporting cell growth. PCL provided easy processability along with favorable biocompatibility and is already being used in various biomedical devices approved by the FDA [154,155,156]. 

**Figure 6:**
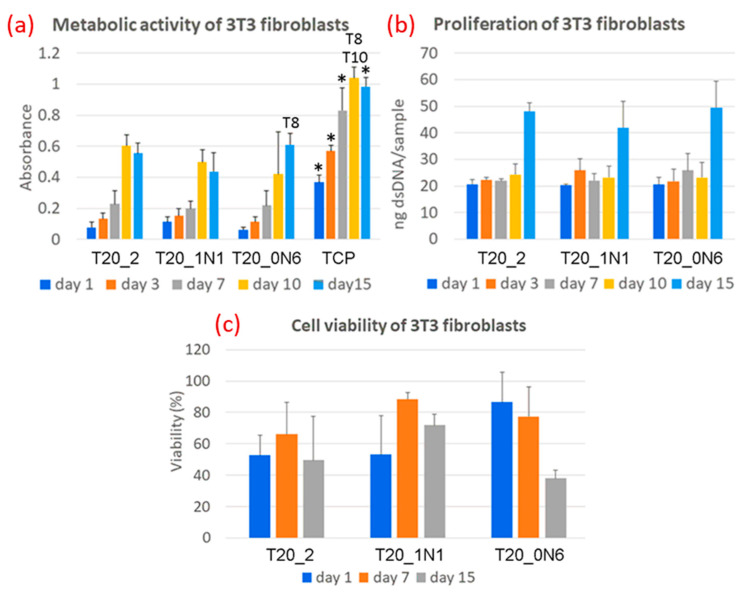
Biocompatible assays carried out on 3T3 fibroblasts immobilized on pristine PCL and TiN NPs-functionalized PCL scaffolds at various concentrations of NPs: (**a**) metabolic activity measured using the MTS assay; (**b**) proliferation using dsDNA assay; and (**c**) viability using live/dead assay. Tissue culture plastic (TCP) was chosen as a reference to provide the highest absorbance in MTS test. * refers to the statistical difference related to all other samples. No significant differences among scaffolds were observed in both cell proliferation and cell viability tests. In the statistics in (**a**), T8 and T10 in the above columns display statistical differences between groups T20_1N1 or T20_0N6, respectively. All assays show results as a mean and standard deviation. Reproduced from Ref. [157].

After the optimization of electrospinning parameters, functionalized nanofibers were obtained, as shown in [157], containing TiNPs fabricated using the laser ablation procedure described [99]. A high concentration of 20% (*w*/*v*) PCL in dichloromethane/acetone (3:2) (*v*/*v*) provided bead-free and smooth nanofibers (400 nm to around 1 micron). The presence of TiN NPs in the nanofibers’ matrix led to a slight decrease in both the degradation initiation temperature and the temperature at which the maximum mass loss takes place. Similar behavior was observed in DSC where pristine PCL nanofibers had a slightly lower temperature corresponding to the melting temperature of nanofibers (T_m_). TiN NPs seemed to act as heating spots in the nanofibers. However, no drastic changes were observed with a changing concentration of TiN NPs themselves in a nanofiber matrix [157]. Finally, biological compatibility tests were performed to test cytotoxicity, adhesion, and metabolic activity using mouse 3T3 fibroblasts as a standard cell line, as shown in Figure 1. At day 15, higher absorbance directly related to metabolic activity was observed. The proliferation of cells observed through the amount of dsDNA, after remaining stable until day 10, increased significantly on day 15 for all samples. Cells showed a good adhesion to scaffolds; however, there are still further improvements required in nanofibers’ surfaces (Figure 6) [157]. In the end, the viability of cells measured through live/dead staining showed no statistical difference. Further long-term in vitro tests using better-developed nanofibers would be needed to better understand the behavior of cells with PCL nanofibers functionalized with TiN NPs.

The presented studies have shown the immobilization of inorganic nanoparticles in nanofibers to fabricate hybrid materials provided a bouquet of unique properties. These characteristics could potentially be used in biomedical applications such as wound dressing, stent coating, cosmetics, nerve guide conduits, drug delivery, etc., with improved antibacterial and mechanical properties. Further, such hybrid structures could be useful for providing a plasmonic effect, photothermal therapy, and improved stability and electrochemical behavior of separators used in batteries. 

## 4. Potential Applications of Hybrid Multifunctional Nanofibers

### 4.1. Nanofiber Application as a Tissue-Engineering Platform

Tissue-engineering approaches appear highly promising for the regeneration of injured tissues [158]. In dentistry, we are looking forward to the technology that allows for the regeneration of tissues such as the periodontal ligament, enamel, dentin, and alveolar bone [159,160]. Tissue engineering combines three critical components: scaffolds, mesenchymal stem cells (MSCs), and growth factors, and seems to be an auspicious approach in dentistry [158,161]. In tissue engineering, scaffolds are used as substitutes for damaged tissue and act as a support for stem cell migration, proliferation, and differentiation. A scaffold must be designed with appropriate biocompatibility, biodegradability, architecture, and mechanical properties to promote the formation of a natural, extracellular matrix [158,161,162]. Recently, hybrid nanofibers, including coated nanofibers, have attracted the attention of investigators since they have shown to promote stem cells’ adhesion, growth, and differentiation into functional cells [160,161,162]. However, in vitro and in vivo experimental studies which described the biological effect of the nanofibrous scaffold with loaded MSCs in bone-tissue engineering are limited [163,164,165]. The use of human dental pulp stem cells (hDPSCs)-derived exosomes encapsulated in triblock poly(lactic-co-glycolic acid) (PLGA), poly(ethylene glycol) (PEG), and PLGA-PEG-PLGA microspheres incorporated into a nanofibrous poly-L-lactide acid (PLLA) scaffold in bone repair defect was proven to be an effective agent in alveolar bone defect regeneration [163]. PLLA combined with hDPSCs induced new bone formation and angiogenesis, leading to bone-tissue regeneration. Scaffolds containing exosomes from mineralizing DPSCs showed high collagen l-rich matrix, new bone tissue, and integration with the host tissue [163]. Interesting results were reported by Malek-Khatabi et al. [164], who evaluated the effects of the microfluidic-assisted synthesis of plasmid DNA (pDNA) encoding human bone morphogenic protein-2 (BMP-2)-based chitosan nanocomplex platforms for bone-tissue engineering. The nanocomplexes were immobilized on a nanofibrous PCL scaffold functionalized with metalloprotease-sensitive peptides. The implantation of MSCs loaded on PCL membranes in a rat calvarial defect model demonstrated a significant increase in the regenerated bone volume. It was found that this composite induced the formation of more dense, bone-like structures [164]. Additionally, Wang et al. [165] developed a hybrid nanofibrous scaffold with poly(lactide-co-ε-caprolactone) (PLCL) modified by silk fibroin. Silk fibroin/PLCL nanofibrous scaffold facilitated the human adipose-derived stem cells (hADSCs) proliferation and osteogenic differentiation. Silk fibroin/PLCL scaffold loaded by hADSCs was implanted in a rat model with critical-sized calvarial defects. The results showed that scaffold with loaded hADSCs enhanced bone regeneration, increased new bone areas, and improved bone mineral density [165]. The authors postulated that the SF/PLCL nanofibrous scaffold holds great potential in bone-tissue regeneration [165]. 

Recently, gene therapy using nanofibers as a drug-delivery system was reported by Qi et al. Hybrid PLGA/gelatin nanofibers loaded with microRNA-181a/b, induced osteogenic differentiation of adipose-tissue derived MSCs in vitro [166]. The functionalization of nanofibers can be performed on the surface, using adhesion of bioactive molecules or platelets [167] and liposomes [168], which can be incorporated either in the core of core/shell fibers, or in the blend composite material (e.g., PVA). Organic solvents may negatively influence bioactivity of bioactive substances; therefore, core-shell electrospinning where hydrophilic polymers such as PVA is in the core is preferred [168,169]. However, other methods, e.g., needleless electrospinning, can also lead to the production of core-shell nanofibers when using an emulsion system for the preparation of nanofibers [169,170,171]. On the other hand, water soluble polymers, e.g., PVA, PEO, PEG, gelatine, hyaluronic acid, and collagen are often used as blends or in composite scaffolds [172,173,174,175]. Bovine serum albumin (BSA)-BMP-2/dexamethasone-loaded core/shell poly(l-lactide-co-caprolactone) (PLLACL) and PLLACL/collagen nanofibers showed an improved osteogenic differentiation of human mesenchymal stromal cells which was accompanied with the controlled release of both BSA-BMP-2 and dexamethasone, while blend nanofibers showed a burst release of dexamethasone [172,173,174]. In addition, PLLA nanofibers loaded with BMP-2, which were prepared from emulsion using electrospinning, stimulated osteogenic differentiation of MSCs in vitro [176,177,178]. Collagen nanofibers with HA nanoparticles loaded with vancomycin or gentamicin released higher concentrations of antibiotics for 21 days compared to collagen. Moreover, the degradation of vancomycin was slowed down [179]. PVA nanofibers loaded with platelet lysate showed stimulation of growth of fibroblasts, keratinocytes, endothelial cells, or maturation of keratinocytes [172]. Interestingly, we have observed that 3D PCL microfibers prepared by centrifugal spinning showed a better proliferation of fibroblasts while 2D core/shell nanofibers prepared by needleless emulsion electrospinning showed a higher proliferation of keratinocytes [169]. Polycaprolactone/gelatin/hyaluronic acid nanofibers were developed to mimic a glioblastoma tumor extracellular matrix [152]. In another study, electrospun PCL membranes blended with hydroxyapatite (HA) were developed, and its potential in differentiating inflamed dental pulp stem/progenitor cells (IDPSCs) into odontoblasts was evaluated [180]. The results showed that fluorapatite coating on the electrospun PCL nanofiber surface facilitated adhesion, proliferation, and differentiation of DPSCs to odontoblasts and might be used as a tool in the therapy of bone damage [180]. Another artificial biomaterial, polyhydroxybutyrate (PHB)/chitosan/nanobioglass (nBG) nanofiber scaffold with seeded human exfoliated deciduous stem cells (SHEDs), has been tested in regenerative dentistry [181]. It was found that nanofibrous scaffold promote SHEDs proliferation and differentiate into odontoblast-like cells [181]. The study revealed that genes’ expression of dentin sialophosphoprotein (DSPP), collagen type I, alkaline phosphatase (ALP), and BMP-2 significantly increased compared to the scaffold as the control group. The results indicated that this scaffold can be used as a suitable substrate to apply in dentin tissue engineering [181]. Most of the existing studies concerning the development of novel therapeutic approaches showed that combining stem cells with biomaterial scaffolds serves as a promising strategy for engineering tissue. 

**Figure 7:**
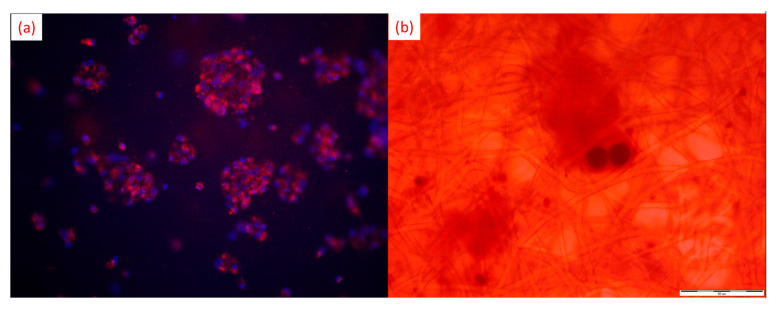
(**a**) Images of hDPSCs grown onto nanofiber scaffold for seven days demonstrate live hDPSCs forming colonies on PLCL surface confirmed by PKH26 red and DAPI staining (magnification ×400); (**b**) osteogenic differentiation of hDPSCs grown on PLCL stained by Alizarin Red S confirmed mineral deposits on PLCL fibers (Scale bar = 50 µm).

A recent experimental study showed that a hydrolytically modified poly(L-lactide-co-caprolactone) (PLCL) electrospun scaffold revealed suitable parameters for human dental pulp stem cells (hDPSCs) growth and differentiation towards osteoblasts [182]. It was found that the porosity and nanofibers’ size were appropriate for hDPSCs proliferation and osteogenic differentiation. It was observed that the loss of mass fibers and increased surface roughness could be beneficial for the biological behavior of hDPSCs. As presented in Figure 7a, hDPSCs grown onto a PLCL scaffold stained by PKH26 Red lipophilic membrane dye showed high proliferation activity and osteogenic potential confirmed by Alizarin Red S staining (Figure 7b).

Advances in tissue engineering need to focus on combinations of dental stem cells with nanofibrous scaffolds, which will induce the appropriate microenvironment and enhance the regenerative potential of dental stem cells.

### 4.2. Nanofibers for Drug-Delivery Applications

**Figure 8:**
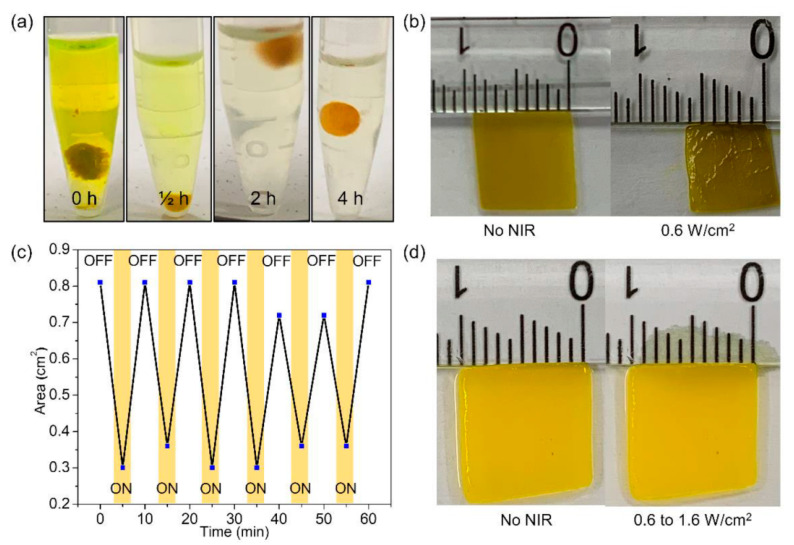
(**a**) Heat treatment of nanofiber under 160 °C. (**b**) Digital images of the area changes of the whole nanofiber containing gold nanorods (GNRs) upon irradiation of NIR light. (**c**) Change in area of the whole nanofiber containing GNRs as a function of cycles of temperature alternation upon the NIR irradiation. (**d**) Digital images of the area of the whole nanofiber without GNRs in the presence and absence of NIR light irradiation. Reproduced from Ref. [188].

Manufacturing of medical devices is a very promising application of composite nanofibers. Their main functions are drug delivery, used to manufacture a controlled drug-release system, bone regeneration, wound healing, and for antibacterial and antitumor applications. Ideal drug-delivery systems (DDS) should release drugs through a specified time frame [183,184,185], not exceeding the toxic concentration of the drug. When the drug concentration is lower than the therapeutic limit, the DDS function is ceased. The main advantage of the DDS is releasing the drug precisely in the place where it is needed, saving the whole body from side effects. This lowers the amount of the drug necessary in DDS by two to three orders of magnitude, which is especially useful for expensive and/or toxic drugs. The main disadvantage of the DDS is the necessity of their implantation and the very low drug load in the system. Only potent therapeutic molecules can be loaded in DDS. The use of inexpensive, anti-inflammatory substances in such forms seems disputable. Different methods of the manufacturing of medical devices based on nanofibers are applied, usually involving nanofibrous mats postprocessing [186,187]. Due to the facility of incorporation of therapeutics in the different forms, e.g., hybrid materials, it grabs the fast-growing attention of medical researchers seeking to find commercializable platforms for on-demand drug delivery. Such a system owning the possibility to perform a drug burst release initiated by a specific signal was proposed by Singh et al. [188]. The authors electrospun a solution of poly(N-isopropylacrylamide) (PNIPAM) hydrogels containing gold nanorods and the anticancer drug known as camptothecin (Figure 8). The authors used a glycidyl derivative of oligomeric silsesquioxane for thermal cross-linking to render the polymer water-insoluble. After water swelling, the polymer mats were tested on brain cancer; specifically, on U-87 MG cell lines. A near-infrared light signal triggered drug expulsion from the mats. Pulsed drug elution, its anticancer cells activity, and swelling–deswelling behavior were evaluated. Pulsed drug release due to sequential laser impulses, nontoxicity of the nanofibers, and infrared light to cells were also proven.

Zhong et al. [189] prepared an adenosine-loaded nanofibrous mat in vivo to assess its utility in bone regeneration in a critical-sized rabbit cranial defect. Electrospun nanofibers of poly(3-hydroxybutyrate-co-3-hydroxyvalerate) with adenosine particles showed great tissue biocompatibility and osteogenic potential leading to successful bone regeneration. It was far more pronounced than in a biocomposite of bone marrow stem cells seeded on the nanofibers with no adenosine added. Fu et al. [190] used a poly(D,L-lactide) nanofibrous mat containing amorphous calcium phosphate nanoparticles. The bovine serum albumin served as a protein drug-like substance and lecithin as a biocompatible surfactant. Such produced systems showed fast mineralization in simulated body-fluid acellular conditions. Nanoparticles seeded mineralization and helped to sustain the model drug release. The mat was highly cytocompatible when seeded with osteoblast-like (MG63) cells. This composite nanofibrous drug-delivery system was aimed at bone-tissue engineering. A new bone regeneration scaffold was produced from two polymers—chitosan and collagen—and three types of nanoparticles—graphene, graphene oxide, and hydroxyapatite [191]. The material showed specific bovine serum albumin adsorption and strong antibacterial properties against E. coli and S. aureus. The biocide effect was most pronounced on composites based on graphene oxide, less on graphene composites, and not present on gelatin and chitosan fibers containing only hydroxyapatite. Nanofiber mats electrospun from polyethersulphone containing hydroxyapatite nanoparticles were evaluated acellularly, in vitro on osteoblast (MG-63) cells and in vivo on rabbits’ tibia [192]. The authors found that the presence of nanoparticles increased bioactivity in simulated body fluid. Additionally, cell adhesion and proliferation were found to increase in cellular studies. Nanoparticles caused a decrease in bovine serum albumin absorption and blood coagulation rate. In vivo experiments showed an intense inflammatory response from the nanocomposite. Huang et al. [193] used bone-marrow-derived mesenchymal stem cells to check cellular biocompatibility and MXene titanium carbide (Ti_3_C_2_) activity. Composite nanofibers were produced from a mixture of poly(L-lactide), other polyhydroxyalkanoate, and MXene nanoparticles. The nanocomposite enhanced cells’ differentiation to osteoblasts. 

Haidar et al. [194] used chitosan nanoparticles loaded with atorvastatin suspended in a solution of poly(L-lactide-co-glycolide) (PLGA) that contained α-lipoic acid to produce a dual therapeutic system based on electrospun nanofibers for peripheral nerve-injury treatment. The rat’s sciatic nerve injury model was assessed with motoric functions recovery and ultrastructural and biochemical analyses of regenerated nerve tissue. The system produced proved to be effective when applied immediately after injury. Zhao et al. [195] manufactured a series of vancomycin-containing PCL nanofibrous membranes covering metallic implants for postoperative protection. The materials were evaluated on a rabbit model of a tracheal implant. The drug release was sustained during in vitro tests and proved efficient against methicillin-resistant S. aureus. and S. pneumoniae. The comparison of a proposed implant covering with a commercially available pellosil matrix showed less granulation tissue and less expression of inflammatory reaction markers. Multiwalled carbon nanotubes and tetracycline hydrochloride were embedded onto poly(lactide)/polyvinylpyrrolidone nanofibers by Bulbul et al. [196]. Such prepared composite nanofibers were evaluated as potential DDS. Surprisingly, the authors found no side effects of carbon nanotubes on a culture of human umbilical vein endothelial cells. The authors assessed the tetracycline hydrochloride release profile and found carbon nanotubes to lower burst release. A composite of core-shell nanofibers containing salicylic acid was proposed as a drug-delivery system [197]. The ratio of core fluid—poly(ethylene oxide)/salicylic acid solution to shell fluid—polylactide solution was optimized. A dichloromethane/dimethylacetamide solvent mixture used to prepare both solutions gave porous fibers, while a chloroform/dimethylacetamide mixture gave nonporous fibers, as shown on the SEM images. Porous nanofibers showed stable, sustained drug release higher than nonporous nonwovens. Drug release from porous mats during five days followed the Fickian diffusion mechanism. Cells of 3T3-L1 and CCD-986sk lines were properly attached and spread on porous, hybrid materials, proving their good biocompatibility. Poly(vinyl pyrrolidone) nanofibers containing ursolic acid were proposed as a fast-dissolving drug-delivery system [198]. Solubility tests showed that electrospun composites had the fastest dissolution rate when compared with a pure drug and its mixture with the polymer, proving the potential for the proposed application. Polycaprolactone nanofibers containing multiwalled carbon nanotubes and green tea polyphenols were proposed as an antitumor DDS [199]. The drug release from the composite nanomaterial was assessed via in vitro tests. Cytotoxic activity was tested in vivo on normal osteoblast cells and tumor cells: A549 and PHep G2. Due to cytotoxic and antiproliferative properties against the tumor cells, the composite was a good candidate for anticancer therapy. A composite, nanofibrous system to fight tumor cells circulating in the organism was proposed by Wang et al. [200]. The authors produced a system with doxorubicin connected to gold nanoparticles via acid cleavable linker activated by a higher pH caused by the tumor cells’ metabolism. The nanoparticles were mixed with poly [2-(dimethylamino)ethyl] methacrylate solution and electrospun. Tumor-specific antibodies (anti-EpCAM) were attached to the mat afterward to capture circulating tumor cells on the therapeutic system.

### 4.3. Biosensing Applications of Nanofibers

Nowadays, there is a huge focus on reducing the footprint of the biosensors used for health monitoring purposes. The idea is to develop flexible sensors, which conform to movements of the user with minimal invasiveness. Here, electrospinning has emerged as an ideal platform for the development of such sensors, as they provide a large surface area to volume ratio for sensing activity and the possibility of the inclusion of molecules promoting selectivity, response time, visual cues (colorimeter), and sensitivity along with flexibility/stretchability [201]. For instance, Rani et al. developed a flexible carbon nanofiber membrane decorated with NiMoO_4_ nanoparticles for efficient glucose sensing applications. They obtained carbon nanofibers (CNF) using a two-step process, initially electrospinning polyacrylonitrile (PAN) and then carbonizing scaffold at a high temperature under nitrogen. The immobilization of nanoparticles was performed by in situ growth of NiMoO_4_ on a CNF surface. The resulting nanofibers showed good sensing response in a wide range of glucose concentrations (0.0003–4.5 mM) at a very low limit of detection (LOD) (50 nM) and high sensitivity (301.77 µA mM^−1^ cm^−2^). Nanofibers were also analyzed to characterize their real-life response where their recovery rate was between 97.5–103.2%, similar to commercial glucometers [202]. Another biochemical sensor for the detection of glucose was developed from coelectrospinning of graphene oxide nanofibers blended with polyvinyl alcohol (PVA) by Baek et el. To realize the sensing of glucose, nanofibers were functionalized by AuNP’s coating and modified with Cu nanoflower by using 1% Nafion (sulfonated tetrafluoroethylene-based fluoropolymer-copolymer) in 2-propanol as a binding agent. The group reported a good linear range of glucose detections between the concentrations of 0.001–0.1 mM at LOD of 0.018 µM [203].

Ozoemena et al. presented a sensor for the detection of dopamine using nanofibers made from onion, such as carbon (OLC) blended with PAN, to enhance electrospinnability. The resulting nanofibers were carbonized to obtain pristine OLC nanofibers. Here, OLC nanofibers displayed the best surface area and pore size during morphology analysis. OLC nanofibers were effective in dopamine sensing with 1.42 µM LOD and 0.31 µA µM sensitivity [204]. These examples show the effectivity of nanofibers for sensing application. Moreover, it highlights the versatility of electrospinning, allowing the processing of diverse materials by blending with polymers with better electrospinnability. Highlights of nanofibers systems with remarkable characteristics and applications are summarized in Table 1. 

## 5. Prospects and Challenges

**Figure 9:**
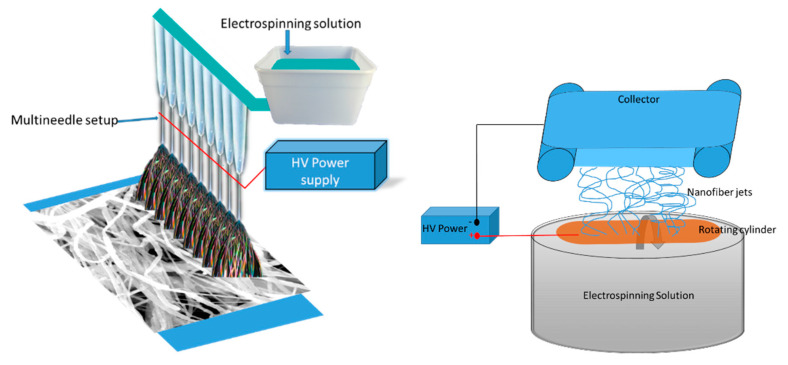
Schematic representation of multiple needle and needleless electrospinning set up for generating higher yields.

Nanofiber systems are revolutionizing and advancing in every possible domain. Nevertheless, there are several reservations when it comes to real life applications of nanofibers. Several challenges are holding back the potential of nanofibers, including yield, cost effectiveness, reproducibility, and inconsistent quality, especially at the commercial scale. Fortunately, many researchers are working in conjunction with industries to tackle some of these challenges. For instance, by applying new approaches for large-scale fabrication and providing novel nanofibers systems, the upscalability of nanofiber production has improved. Emerging production techniques of nanofibers offer a promising potential for obtaining consistently significant yields. The standard needle-based spinning techniques enable the production of only ~0.2 g h^−1^. However, there are several innovative principles for improving yield: (i.) multineedle electrospinning utilizes a high number of needle-based emitters arranged into arrays; (ii.) needleless electrospinning utilizes special emitters enabling the self-arrangement of multiple fiber jets on their surface (Figure 9) [206,207,208]. Needleless electrospinning was also shown to produce drug-delivery nanofiber systems [169,170,171] and nanoparticle-loaded nanofibers [209]. In addition, the surface of needleless, electrospun nanofibers were shown to be functional with antibodies. Needleless electrospinning produced PVA nanofibers, which were functionalized by PEG-biotin linkers and subsequently functionalized by antibodies and enzymes [210]. The system showed functional proteins on the surface and controlled degradation based on functionalization degree. Similarly, chitosan nanofibers prepared by needleless spinning were functionalized by anti-CD44 antibodies, resulting in enhanced osteogenesis in vivo [211]. Thus, needleless electrospinning can produce nanofibers on an industrial scale, and helps with the translation of the functionalization system to clinical practice.

## 6. Conclusions

Advances in the fabrication of hybrid nanofibers open new avenues for the next generation of multifunctional nanocompartments via utilization of novel combinations of functional nanomoieties. In this review, the fabrication, characterization, and optimization of nanofibers containing unique modalities, such as naturally occurring biocompatible and biodegradable polymers, are shown to present their own challenges. Combining vanities of polymers along with functional agents to form multifunctional nanocompartments demand new strategies. Herein, multiple such hybrid nanofibers have been presented, where the combination of a polymeric matrix and functional agents are utilized to generate multifunctional nanocompartments with unique collective properties. Co-electrospinning and template spinning approaches are merging polymers which possess excellent electrospinnability, higher processability, and immobilization with functional agents, such as laser ablated NPs, providing exceptional surface properties. The review presents these strategies which allow for the fabrication of stable, functional hybrid nanofibers. The generated nanofibers possess unique collective properties and facilitate a platform for a wide range of applications, such as biosensors, drug delivery, theranostics, tissue engineering. For instance, the immobilization of TiNPs within PCL nanofibers provide a potential platform for cancer cell theranostics and tissue engineering. These applications demand the implementation of novel combinations of hybrid nanocompartments, leading to further development and optimization of electrospun, hybrid nanofiber techniques. Such hybrid nanofiber systems comprising functional agents such as drugs, growth hormones, nutrients, nanoparticles, perovskites, etc., can be employed for tissue engineering and biomedical applications, among others, even though the fabrication of these hybrid nanofibers at the industrial scale is still at a very basic juncture. With extensive research, the potential of hybrid, multifunctional nanocompartments will be realized sooner than predicted.

## Figures and Tables

**Table 1:** Summarized nanofiber systems with remarkable characteristics and applications.

Nanofiber Matrix	Functional Materials	Application
Poly(ε-caprolactone) (PCL)	Poly(ethylene glycol) modified with carboxylic acid spiropyran	Sensors: Detections of metal ions such as Mg^2+^, Ca^2+^, Zn^2+^, Cd^2+^, La^3+^, and Er^3+^. Nanofibers with metals ions absorbed demonstrated orange fluorescence when exposed to UV rays [25].
Fibrinogen: poly(ε-caprolactone) (PCL)	Fibrinogen	Wound dressing: Biocompatible nanofibers with improved morphology and mechanical properties for creating organoids or dressings, and drug delivery [28].
Elastin: poly-lactic-co-glycolic acid (PLGA)	Elastin	Tissue engineering: Facilitation of epithelial cell self-organization into cell clusters. Useful for regenerative therapies for salivary glands and other epithelial organs [30].
Silk fibroin: poly(L-lactic acid-co-ε-caprolactone) (PLCL)	Silk fibroin	Tissue engineering: Proliferation and culture of rabbit conjunctival epithelial cells with reduced expression of inflammatory mediators. Scaffolds for conjunctival reconstruction [31].
Hyaluronic acid (HA): poly(vinyl alcohol) (PVA)	Naproxen	Drug delivery: Controlled drug-delivery agents with stabilized release profile maintained over several days; stable HA nanofiber structure [33].
Hydroxypropyl-beta-cyclodextrin	Ibuprofen	Drug delivery: Fast-action oral drug-delivery systems, water soluble. Polymer-free electrospinning system [34].
Poly(e-caprolactone) (PCL): poly(3-hydroxybutyric acid) (PHB)	Hydroxybenzo[a]phenazine pyrazol-5(4H)-one	Drug delivery: Excellent cyotoxicity against MCF-7 and Hep-2 cancerous cell lines. Induction of apoptosis and suppression of proliferation of cancerous cells [36].
Poly(butylene adipate-co-terephthalate) (PBAT)	Nano-hydroxyapatite (nHAp)	Tissue engineering: Biocompatible scaffolds for improving bone volume, stiffness, and promoting bone repair [46].
Polyvinyl pyrrolidone (PVP)/tetrabutyl titanate (TBT)	TiO_2_ nanoparticles and upconverted NaYF_4_:Yb/Tm@NaYF_4_ nanoparticles	Catalysis: Excellent photocatalytic activity, enhanced UV emission under irradiation of Near IR light [53].
Polyurethane (PU)	Superparamagnetic iron oxide nanoparticles (SPIONs)	Therapy: Nanofibers show progressive heat-generation capacity with increasing magnetic nanoparticle concentrations. Heat-generating substrate for localized hyperthermia cancer therapy [56].
Polyvinyl-alcohol(PVA)	Titanium dioxide (TiO_2_)	Solar cells: Light-scattering layer, increase in power conversion, and charge-collection efficiency [64].
Poly(ethylene terephthalate) (PET)	-	Filters: Nanofiber filtration membrane with 98% efficiency trapping particles with a size of up to 120 nm and water permeation capacity of 94% [66].
Poly(vinylidene fluoride) (PVDF):poly(methyl methacrylate-random-perfluorodecyl methacrylate), P(MMA-r-FDMA)	Perfluorodecyl methacrylate	Filters: Nanofiber with excellent mechanical strength suitable for separation of oil and water. Fouling resistant, hydrophobic, and superoleophilic membrane [68].
Poly(L-lactide-co-glycolide) (PLGA)	Metal Halide Perovskites	Tissue engineering: Perovskite-based nanofibers mimicking mechanical properties of skin. Promotes proliferation of human dermal fibroblasts; antimicrobial [119].
Polyacrylonitrile	Graphene quantum dots	Sensors: Fluorescence sensors for free chlorine detection [205].
Poly(e-caprolactone) (PCL)	Bone morphogenic protein-2 (BMP-2), heparin (Hep)	Tissue engineering: Scaffolds with enhanced osteogenicity and proliferation for ligament regeneration and bone integration [159].
Poly(L-lactic acid) (PLLA)	Stem cell-derived exosomes microspheres	Tissue engineering: Controlled delivery of the exosomes to stimulate bone tissue neogenesis [163]
Poly (lactic-co-glycolic acid) (PLGA)	MicroRNAs	Tissue engineering: Using gene therapy with scaffolds promoting osteogenic differentiation capacity of the human (adipose-derived mesenchymal stem cells) AT-MSCs [166].
Poly(e-caprolactone) (PCL)	Hydroxyapatite	Tissue engineering: Promoting cell adhesion and odontogenic differentiation of inflamed dental pulp stem cells (IDPSCs) [180].
Polyhydroxybutyrate (PHB):Chitosan	Nano-bioglass (nBG)	Tissue engineering: Promoting proliferation and differentiation of stem cells obtained into odontoblast-like cells. Substrate for dentin tissue engineering [181].
Poly (N-isopropylacrylamide) (PNIPAM)	Gold nanorods	Drug-delivery system: Light-sensitive, on-demand drug-delivery system, capable of targeted drug delivery [188].
Poly lactic-co-glycolic acid (PLGA)	Atorvastatin loaded chitosan NPs.	Drug-delivery system: Enhance recovery and regeneration capacity of neural sensory and motor system through controlled and fast-action drug release [194].
Poly(e-caprolactone) (PCL)	Vancomycin	Drug delivery system: PCL/VA film-coated metallic stent antimicrobial activity, drug carrying capacity, and structural support [195].
Poly (lactic acid) (PLA):polyvinylpyrrolidone (PVP):carbon nanotubes	Tetracycline hydrochloride	Drug-delivery system: Cytocompatible nanofibers with improved mechanical properties, controlled drug release-profile [196].
Carbon nanofiber	NiMoO_4_ NPs	Sensors: High-performance glucose sensors [202].
Graphene oxide: poly(vinyl alcohol) (PVA)	Copper-nanoflower decorated gold NPs	Sensors: Monitor glucose levels in biofluids [203].
Polyacrylonitrile (PAN): Carbon	Onion-like carbon composites	Sensors: Biosensors for detection of dopamine [204].
